# Tenosynovial giant cell tumors in unusual locations detected by positron emission tomography imaging confused with malignant tumors: report of two cases

**DOI:** 10.1186/s12891-016-1050-7

**Published:** 2016-04-26

**Authors:** Akihiko Takeuchi, Norio Yamamoto, Katsuhiro Hayashi, Shinji Miwa, Masayuki Takahira, Kiyokazu Fukui, Taku Oikawa, Hiroyuki Tsuchiya

**Affiliations:** Department of Orthopaedic Surgery, Graduate School of Medical Science, Kanazawa University, 13-1 Takara-machi, Kanazawa-shi, 920-8641 Ishikawa-ken Japan; Department of Ophthalmology, Graduate School of Medical Science, Kanazawa University, 13-1 Takara-machi, Kanazawa-shi, 920-8641 Ishikawa-ken Japan; Department of Orthopaedic Surgery, Kanazawa Medical University, 1-1 Daigaku, Uchinada-machi, Kahoku-gun, 920-0293 Ishikawa-ken Japan; Department of Respiratory Medicine, Kanazawa Medical University, 1-1 Daigaku, Uchinada-machi, Kahoku-gun, 920-0293 Ishikawa-ken Japan

**Keywords:** Tenosynovial giant cell tumor, FDG PET/CT, Biopsy, Malignant tumor

## Abstract

**Background:**

A tenosynovial giant cell tumor (T-GCT) is a benign synovial tumor arising from the synovium, bursae, or tendon sheath. It can be intra- or extra-articular and localized or diffuse. Diffuse T-GCT is considered as a locally aggressive. Positron emission tomography (PET) with fluorine-18 fluorodeoxyglucose with computed tomography (FDG PET/CT) is widely used to differentiate malignant from benign tumors and to detect distant metastasis. However, FDG PET/CT is limited by false-positive findings. In this study, we present two cases of T-GCT that developed in unusual locations and were confused with malignant tumors. The final diagnoses were histologically confirmed as T-GCTs.

**Case presentation:**

Case 1. A 45-year-old Japanese female presented with a left choroidal melanoma and an abnormal lesion adjacent to the first cervical (C1) lamina confirmed by a PET scan (maximum standardized uptake value [SUVmax] = 9.9 g/ml). MRI of the neck also detected a soft tissue mass (14.6 × 7.7 × 7 mm) adjacent to the C1 lamina. The choroidal melanoma was treated by heavy carbon ion radiotherapy. Although the size of the C1 soft tissue tumor remained unchanged, a CT-guided biopsy confirmed the diagnosis of the neck mass as a T-GCT.

Case 2. A 15-year-old Japanese male with multiple type 1 neurofibromatosis presented with a soft tissue mass (26.1 × 24.7 × 11.5 mm) of the extra-articular hip joint that was coincidentally detected by FDG PET/CT during examination of a mediastinal soft tissue mass. SUVmax of the mediastinal lesion was 2.6 g/ml and of the hip lesion was 12.8 g/ml. Thus, differentiation from a malignant tumor, such as a malignant peripheral nerve sheath tumor, was necessary. An open biopsy was performed, and the frozen section was diagnosed as T-GCT. The tumor was excised, and the final histological diagnosis confirmed T-GCT.

**Conclusion:**

T-GCT can show high FDG uptake, which might be confused with malignancy. Although MRI findings and location might help in the diagnosis of a T-GCT, careful assessment is mandatory, especially in unusual locations.

## Background

A tenosynovial giant cell tumor (T-GCT) is a benign, synovial tumor arising from the synovium, bursae, or tendon sheath. It can be intra- or extra-articular and localized or diffuse. A localized extra-articular T-GCT is synonymous with a giant cell tumor of the tendon sheath; it usually occurs in the hand (other sites include the wrist, ankle/foot, and knee) and is more often seen in women than men [1], with a reported annual incidence of about one in 50,000 [2]. Diffuse T-GCT is considered as a locally aggressive and predominantly involves the knee, followed by the hip, ankle, elbow, and shoulder [3]; it has a reported annual incidence of approximately two cases per million, mostly younger than 40 years of age, with no gender predilection [4]. Unusual sites of T-GCTs include extra-articular neck and hip [5, 6].

Positron emission tomography (PET) with fluorine-18 fluorodeoxyglucose (FDG) with computed tomography (FDG PET/CT) is widely used to differentiate malignant from benign tumors and to detect distant metastasis [7, 8]. However, as on FDG is not cancer-specific, false-positive findings of benign diseases have been reported in active inflammation or infection [9–11]. Schulte et al. reported a 15 % false-positive rate of malignancy among 200 tumor and tumor-like lesions of the bone [12]. The appearance of T-GCT in FDG PET/CT was not fully reported because this modality is not routinely used. However, there are reports of varied FDG uptake (maximum standardized uptake value [SUVmax] = 3.43–25 g/ml) in T-GCTs, which mimic malignant tumors [13–16].

In this article, we discuss two cases of T-GCT that developed in unusual locations and were initially thought to be malignant tumors on FDG PET/CT, but were histologically confirmed as T-GCT.

## Case presentation

### Case 1

**Fig. 1:**
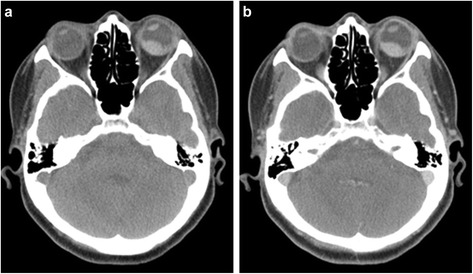
Noncontrast CT detected a high attenuation mass (16 × 11 mm) in the left choroid plexus (**a**) that showed enhancement after contrast administration (**b**)

**Fig. 2:**
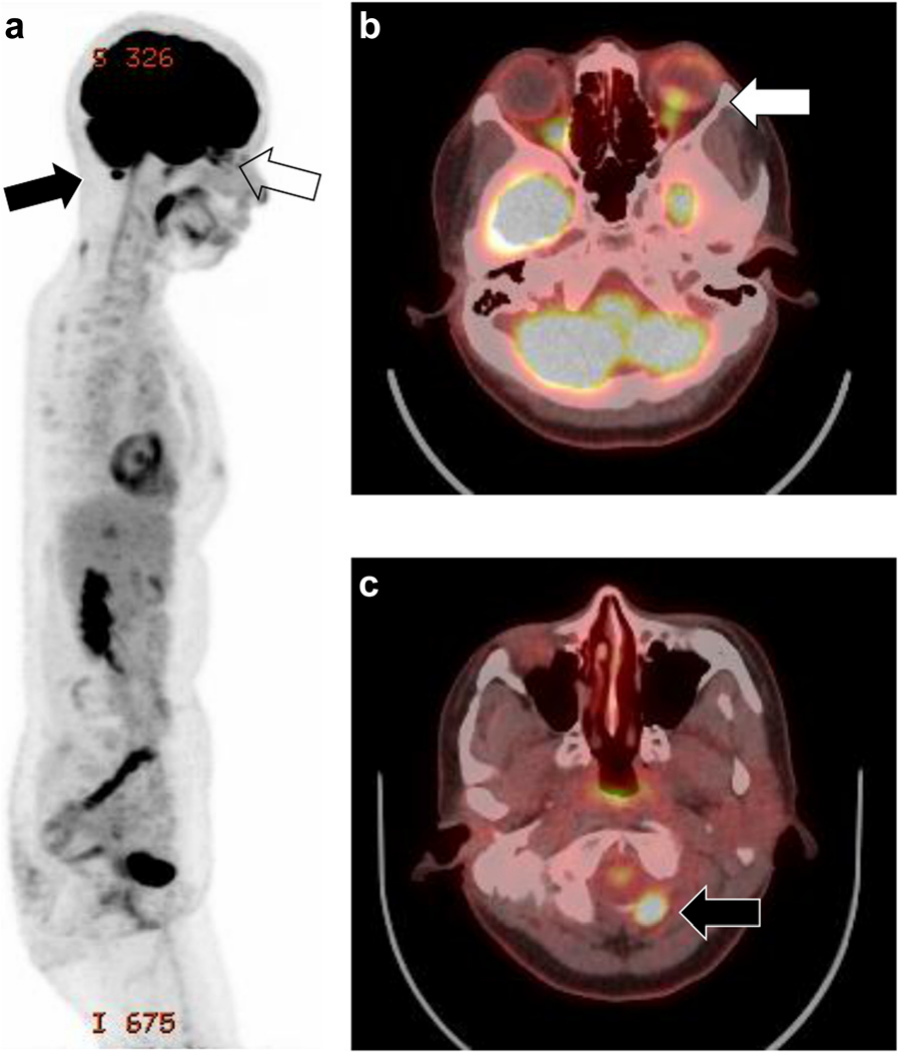
FDG PET/CT sagittal image (**a**) revealed two focal regions of increased uptake in the eye (*white arrow*) and posterior neck (*black arrow*). Axial image (**b**) showed increased FDG uptake in the eye (*white arrow*) and the choroid plexus of the left eye (SUVmax = 3.8 g/ml). Axial image (**c**) of the first cervical (C1) spine showed increased uptake (*black arrow*) adjacent to the C2 lamina (SUVmax = 9.9 g/ml)

**Fig. 3:**
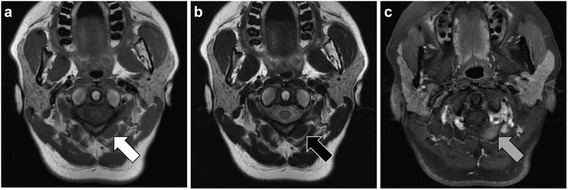
Axial MRI of a soft tissue mass (14.6 × 7.7 × 7 mm) adjacent to the C1 lamina showed isointensity on a T1WI (*white arrow*) (**a**), low intensity on a T2WI (*black arrow*) (**b**), and enhanced gadolinium uptake (*gray arrow*) (**c**)

**Fig. 4:**
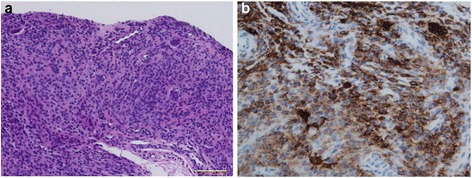
Biopsy specimen showed small mononuclear stromal cells with stromal fibrosis, including formation of a hyalinized collagen matrix and a small area of multinucleated osteoclast-like giant cells (hematoxylin and eosin staining; scale bar, 100 μm) (**a**). Immunohistochemical analysis showed strong staining for CD68 (**b**)

A 45-year-old Japanese woman presented with an abnormal lesion in her left eye (Fig. 1) in January 2015. FDG PET/CT detected mild FDG uptake in the left choroidal plexus lesion (SUVmax = 3.8 g/ml) and an intensely hypermetabolic extra-articular soft tissue density lesion adjacent to the first cervical (C1) lamina (SUVmax = 9.9 g/ml) (Fig. 2). *N*-isopropyl-*p*-^123^I-iodoamphetamine single-photon emission computed tomography showed high uptake in the left choroidal plexus lesion; thus, we arrived at a diagnosis of choroidal melanoma. Magnetic resonance imaging (MRI) of the neck detected a soft tissue mass (14.6 × 7.7 × 7 mm) adjacent to the C1 lamina, demonstrating an isointense signal on a T1-weighted image (WI), low signal on a T2WI, and enhanced gadolinium uptake (Fig. 3). She had no symptom related to the neck lesion. The choroidal melanoma was treated by heavy carbon ion radiotherapy from January to March 2015. The size of the soft tissue tumor of C1 remained unchanged, and we performed a CT-guided biopsy to confirm the diagnosis in mid-March 2015. Biopsy specimen showed small mononuclear stromal cells with stromal fibrosis, including formation of a hyalinized collagen matrix and a small area of multinucleated osteoclast-like giant cells (Fig. 4a). Immunohistochemical analysis showed strong staining for CD68 (Fig. 4b). The final histological diagnosis was localized T-GCT. The tumor size remained stable for 6 months after biopsy, and no functional deficit was detected.

### Case 2

**Fig. 5:**
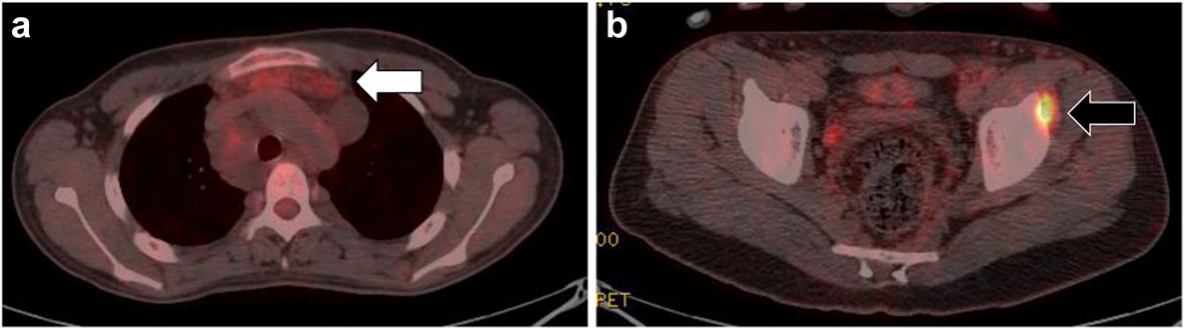
Axial FDG PET/CT image (**a**) showed that the FDG uptake (*white arrow*) in the mediastinal mass was mild or low-level (**a**) and high uptake (*black arrow*) in the extra-articular soft tissue mass of the left hip (SUVmax = 12.8 g/ml) (**b**)

**Fig. 6:**
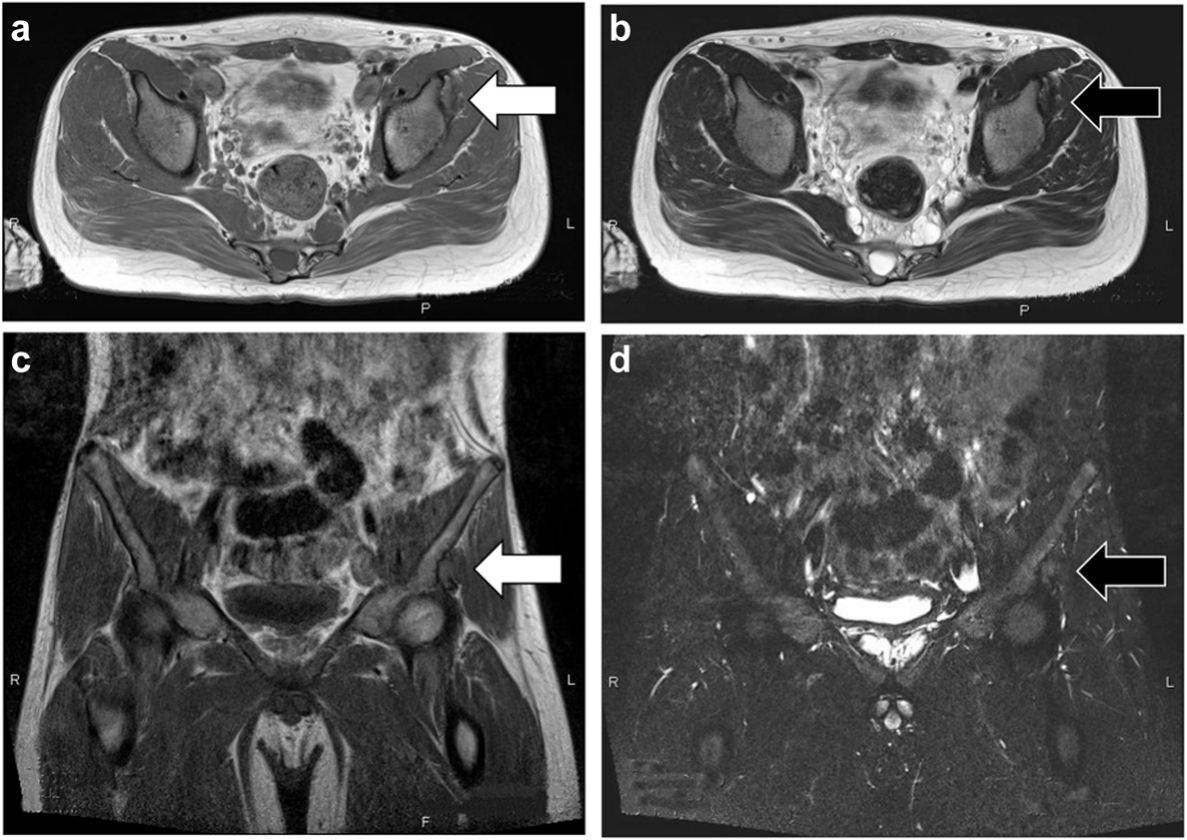
Axial MRI showed a soft tissue mass adjacent to the anterior inferior iliac spine, which was isointense on T1WI (*white arrow*) (**a**) and T2WI (*black arrow*) (**b**). Coronal images showed isointensity in T1WI (*white arrow*) (**c**) and T2WI with fat saturation image (*black arrow*) (**d**)

**Fig. 7:**
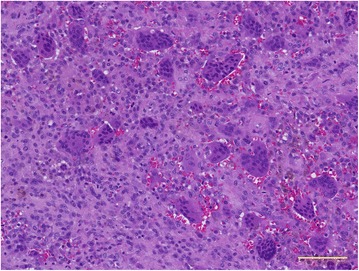
Excised tumor specimen showed small mononuclear stromal cells and multinucleated osteoclast-like giant cells (hematoxylin and eosin stain; scale bar, 100 μm)

A 15-year-old Japanese male was referred to our university hospital for evaluation of an abnormal lesion of the extra-articular hip joint in July 2015. He was previously diagnosed with multiple neurofibromatosis (neurofibromatosis type 1; NF-1) and had a history of cleft palate and hydronephrosis at the age of 7 years. A plain chest radiograph in 2014 had showed mediastinal widening. MRI revealed a soft tissue mass in the mediastinum. In May 2015, FDG PET/CT showed low FDG uptake (SUVmax = 2.6 g/ml) in the mediastinal mass and an intensely hypermetabolic extra-articular soft tissue density lesion in the left hip (SUVmax = 12.8 g/ml) (Fig. 5). MRI detected a soft tissue mass (26.1 × 24.7 × 11.5 mm) located along the proximal indirect tendon of the rectus femoris muscle, demonstrating isointense signals on T1- and T2WIs (Fig. 6). He had no symptom related to the hip soft tissue mass. Because of the previous diagnosis of NF-1, differentiation from a malignant tumor, such as a malignant peripheral nerve sheath tumor, was necessary. A percutaneous core needle biopsy was performed in June 2015 at the previous hospital, however the material was insufficient to define the diagnosis. An open biopsy was performed in July 2015, and the frozen section was diagnosed as T-GCT. Thus, we excised the tumor. Excised tumor specimen showed small mononuclear stromal cells and multinucleated osteoclast-like giant cells (Fig. 7). The final histological diagnosis was localized T-GCT. He showed no functional deficit 2 months after the surgery.

## Conclusions

T-GCT is a benign soft tissue tumor that arises from the synovium, bursae, or tendon sheath that typically contains areas of intermediate and/or low signal intensity on T1- and T2WIs. A T-GCT is usually diagnosed by MRI [17]. In our patients, the MRI findings were compatible with T-GCTs, even though the masses were located in unusual sites. Moreover, FDG PET/CT is not frequently employed for T-GCT, as the findings cannot be confirmed. Hamada et al. analyzed FDG PET/CT images of 56 soft tissue tumors (19 malignant and 37 benign tumors) and found statistically significant difference in the SUVmax between malignant and benign lesions in early scans (5.50 ± 5.32 g/ml versus 3.10 ± 2.64 g/ml). Moreover, the intensities of benign soft tissue lesions were <6.0 g/ml [18]. Broski et al. reported a mean SUVmax of 8.7 (range, 4.0–14.5) g/ml for FDG PET/CT imaging of 14 T-GCTs, and concluded that a T-GCT could be intensely hypermetabolic, mimicking musculoskeletal metastasis [15]. Selby et al. reported a pigmented villonodular synovitis of the shoulder mimicking a metastatic melanoma [16]. Lindkog et al. reported false-positive results of PDG PET/CT in five cases of benign soft tissue lesions, including two T-GCT lesions, and mentioned the importance of understanding the limitations of PET, including false-positive findings of benign lesions [11]. Hamada et al. reported a positive correlation of FDG SUVmax with the expression of GLUT-1 and hexokinase II in 49 musculoskeletal tumors [19]. However, the expression patterns of GLUT-1 and hexokinase II in T-GCTs have not been fully evaluated. Therefore, the mechanism of high uptake of FDG PET/CT in T-GCT remains uncertain. Although FDG PET/CT is a useful imaging modality for patients with a history of malignancy and the demand has been increasing, the chance of detecting benign lesions is also increased. When a T-GCT is detected in a typical location and MRI findings are compatible to T-GTC, careful observation without histological confirmation is reasonable. However, if a suspected T-GCT is detected in an unusual location, a biopsy should be performed for differentiation from a malignant tumor. Recently, there have been new developments in the medical treatment of T-GCT using a selective colony-stimulating factor 1 receptor (CSF1R) kinase inhibitor [20]. FDG PET/CT might be a useful imaging modality in monitoring this treatment [21].

In conclusion, we encountered two cases of T-GCTs that developed in unusual locations, which were confused with malignant tumors. The final diagnoses of these cases were histologically confirmed as T-GCTs. It is important to realize that a T-GCT may show high FDG uptake, which can resemble from a malignant tumor. Although MRI findings and the location might help to diagnose a T-GCT, careful assessment is mandatory.

## Consent

Written informed consent was obtained from Patient #1 and from the parents or legal guardians of Patient #2 for publication of these case reports and any accompanying images. Copies of the written consent forms are available for review from the Section Editor of this journal.

## Availability of data and materials

All the data supporting our findings is contained within the manuscript.

## Abbreviations

T-GCT: tenosynovial giant cell tumor
FDG: fluorodeoxyglucose
PET: positron emission tomography
FDG PET/CT: positron emission tomography with fluorine-18 fluorodeoxyglucose and computed tomography
MRI: magnetic resonance imaging
SUVmax: maximum standardized uptake value
NF-1: neurofibromatosis type 1
CSF1R: colony-stimulating factor 1 receptor
